# Seizures regulate the cation-Cl^−^ cotransporter NKCC1 in a hamster model of epilepsy: implications for GABA neurotransmission

**DOI:** 10.3389/fneur.2023.1207616

**Published:** 2023-06-22

**Authors:** Juan-Manuel Bonet-Fernández, Pedro Tranque, Jose Daniel Aroca-Aguilar, Luis J. Muñoz, Dolores E. López, Julio Escribano, Carlos de Cabo

**Affiliations:** ^1^Neuropsychopharmacology Unit, Research Department, Albacete General Hospital, Albacete, Spain; ^2^Biomedical Instrumentation Service, Faculty of Medicine, University of Castilla-La Mancha, Albacete, Spain; ^3^Department of Medical Sciences, Faculty of Medicine, University of Castilla-La Mancha, Albacete, Spain; ^4^Department of Genetics, Faculty of Medicine/Instituto de Investigación en Discapacidades Neurológicas (IDINE), University of Castilla-La Mancha, Albacete, Spain; ^5^Instituto de Neurociencias de Castilla y León (INCYL), University of Salamanca, Salamanca, Spain

**Keywords:** brain, epilepsy, audiogenic seizures, GASH/Sal, NKCC1, GAD67, hippocampus, hypothalamus

## Abstract

**Background:**

The balance between the activity of the Na^+^/K^+^/Cl^−^ cotransporter (NKCC1) that introduces Cl^−^ into the cell and the K^+^/Cl^−^ cotransporter (KCC2) that transports Cl^−^ outside the cell is critical in determining the inhibitory or excitatory outcome of GABA release. Mounting evidence suggests that the impairment of GABAergic inhibitory neurotransmission plays a crucial role in the pathophysiology of epilepsy, both in patients and animal models. Previous studies indicate that decreased KCC2 expression is linked to audiogenic seizures in GASH/Sal hamsters, highlighting that Cl^−^ imbalance can cause neuronal hyperexcitability. In this study, we aimed to investigate whether the Na^+^/K^+^/Cl^−^ cotransporter NKCC1 is also affected by audiogenic seizures and could, therefore, play a role in neuronal hyperexcitability within the GASH/Sal epilepsy model.

**Methods:**

NKCC1 protein expression in both the GASH/Sal strain and wild type hamsters was analyzed by immunohistochemistry and Western blotting techniques. Brain regions examined included cortex, hippocampus, hypothalamus, inferior colliculus and pons-medulla oblongata, which were evaluated both at rest and after sound-inducing seizures in GASH/Sal hamsters. A complementary analysis of NKCC1 gene *slc12a2* expression was conducted by real-time PCR. Finally, protein and mRNA levels of glutamate decarboxylase GAD67 were measured as an indicator of GABA release.

**Results:**

The induction of seizures caused significant changes in NKCC1 expression in epileptic GASH/Sal hamsters, despite the similar brain expression pattern of NKCC1 in GASH/Sal and wild type hamsters in the absence of seizures. Interestingly, the regulation of brain NKCC1 by seizures demonstrated regional specificity, as protein levels exclusively increased in the hippocampus and hypothalamus. Complementary real-time PCR analysis revealed that NKCC1 regulation was post-transcriptional only in the hypothalamus. In addition, seizures also modulated GAD67 mRNA levels in a brain region-specific manner. The increased GAD67 expression in the hippocampus and hypothalamus of the epileptic hamster brain suggests that NKCC1 upregulation overlaps with GABA release in these regions during seizures.

**Conclusions:**

Our results indicate that seizure induction causes dysregulation of NKCC1 expression in GASH/Sal animals, which overlaps with changes in GABA release. These observations provide evidence for the critical role of NKCC1 in how seizures affect neuronal excitability, and support NKCC1 contribution to the development of secondary foci of epileptogenic activity.

## Introduction

Gamma-aminobutyric acid (GABA) is the main inhibitory neurotransmitter in the CNS. The control of neuronal excitability by GABA is essential in regulating many brain processes, including motor functions, cognition, anxiety and sleep (1). GABA synthesis is mediated by the enzyme glutamic acid decarboxylase (GAD), which exists in two isoforms: GAD67 and GAD65. GAD67, the predominant GAD isoform in the brain, plays a critical role in maintaining basal levels of GABA (2). In consequence, the deletion of the *GAD67* gene *Gad1* leads to a dramatic reduction of 90% in GABA levels within the brain, ultimately resulting in perinatal death (3). GAD67 is expressed throughout the cytosol of neurons and glial cells (4, 5). In GABAergic neurons, GAD67 exhibits widespread expression, while GAD65 is predominantly localized in synaptic terminals (6). Moreover, there is evidence suggesting that GAD67 expression is influenced by neuronal activity and regulated at the transcriptional level, making it a sensitive indicator of changes in GABAergic activity within the brain (4).

GABA binding to its type A ionotropic receptors (GABA_A_Rs) can generate both inward and outward Cl^−^ currents in the postsynaptic neuron, depending on the Cl^−^ electrochemical gradient across the neuronal membrane. The regulation of Cl^−^ ion gradients in neurons is mainly controlled by cation-Cl^−^ cotransporters NKCC1 (Na^+^-K^+^-2Cl^−^) and KCC2 (K^+^-2Cl^−^). NKCC1 is also expressed in astrocytes, where its role in regulating ion concentrations in the extracellular space is crucial for effective neurotransmission. While NKCC1 pumps Cl^−^ into the cell, KCC2 transports Cl^−^ outside. Therefore, the balance between NKCC1 and KCC2 expression significantly impacts ion homeostasis and neuronal excitability (1, 7, 8). Throughout neuronal maturation, the balance between NKCC1 and KCC2 expression changes, resulting in a switch from excitatory to inhibitory GABA neurotransmission. In the developing brain, the elevated expression of NKCC1 causes immature neurons to depolarize in response to GABA release. However, in the adult brain, the neuronal expression of NKCC1 decreases, and KCC2 increases (9), leading to a reduced intracellular Cl^−^. This results in GABA release causing a net efflux of Cl^−^ through GABA_A_R, which hyperpolarizes the neuron and decreases its excitability (7, 10).

Both patients and animal models provide evidence that GABA dysregulation is involved in the development and progression of epilepsy, a condition where the balance between excitatory and inhibitory neurotransmission is disrupted (1, 11). For example, inhibiting GABA release from parvalbumin-expressing interneurons in the mouse hippocampus induces hyperexcitability and spontaneous seizures (12). Many drugs used to prevent or terminate seizures, including benzodiazepines and barbiturates, act by enhancing GABA inhibitory neurotransmission (13). Additionally, upregulation of GAD67 expression has been linked to enhanced GABA inhibitory neurotransmission in brain regions where epileptiform activity is generated, both in human temporal lobe epilepsy (14) and in mouse models of epilepsy (15).

According to the literature, altered function of NKCC1 and KCC2 can lead to increased Cl^−^ influx into neurons, contributing to hyperexcitability and epileptic seizures by reducing the effectiveness of GABA-mediated inhibition. This is supported by studies showing upregulated NKCC1 expression in patients with refractory epilepsy (16). The effectiveness of the NKCC1 inhibitor bumetanide in decreasing seizure activity in a rat model of hypoxic neonatal epilepsy further supports this notion (17). Therefore, targeting Cl^−^ cotransporters may be an effective therapeutic strategy for epilepsy. Additionally, NKCC1 and KCC2 have been implicated in a range of neurological diseases beyond epilepsy, including Down syndrome, Parkinson's disease, Alzheimer's, Huntington's disease, and neuropsychiatric disorders such as schizophrenia (18). However, further research is required to fully understand the role of these ion cotransporters in neuropathology.

The hamster strain GASH/Sal is a natural model for audiogenic seizures, where altered GABA signaling plays a key role (19–21). The inferior colliculus is a major center for auditory processing, where GABA is the central inhibitory neurotransmitter; it is the generally accepted initiation site for all audiogenic models. As seizures progress, they involve primary auditory nuclei in the brainstem, such as the cochlear nucleus (22). A previous study has shown that repeated seizures can alter the expression pattern of KCC2 protein in the brain of GASH/Sal animals (23). Specifically, KCC2 expression decreases in brain areas such as the inferior colliculus and hippocampus, while it increases in the medulla oblongata. This widespread disruption of KCC2 expression in the GASH/Sal brain makes this audiogenic model a valuable tool for investigating the link between dysregulation of Cl^−^ cotransporters and seizure induction. However, the expression profile of NKCC1 in this hamster model has not yet been investigated.

This study aimed to examine the link between seizures and dysregulated NKCC1 expression in GASH/Sal epileptic hamsters. Brain NKCC1 expression in the GASH/Sal hamster was compared to that of wild-type animals, both at rest and after repeated sound-induced seizures. GAD67 expression was also analyzed as an indicator of changes in the GABAergic system. The results obtained show a significant increase in NKCC1 expression in response to seizures and underline the overlapping of NKCC1 changes with enhanced GAD67 expression, indicative of increased GABA release in specific brain areas. Overall, these findings support the idea that dysregulated NKCC1 expression is implicated in epileptogenic activity.

## Materials and methods

### Animals

The experimental animals were handled and cared for according to the guidelines of the Spanish (RD 53/2013) directives under the supervision of the University of Castilla-La Mancha Animal Care Committee, which were in accordance with the Declaration of Helsinki and the Guidelines of the Directive 2010/63/EU of the European Parliament and of the Council. All efforts were made to avoid unnecessary animal suffering and to reduce the number of animals used. The epileptic hamster groups consisted of three-month-old male seizure-prone hamster belonging to the GASH/Sal strain. This strain derives from one original epileptic hamster which appeared spontaneously at the University of Valladolid and gave rise to the first seizure-prone hamster strain called GPG:Vall (19). The hamsters in this study were obtained from the inbred strain maintained at the animal facility of the University of Salamanca (USAL, Spain) and were housed during the experiments at facilities of the Research Department of the Albacete General Hospital, Spain. The GASH/Sal strain exibits the following behavior after sound stimulation: a post-stimulus latency period (phase 1), wild running (phase 2), tonic-clonic seizures (phase 3), and stupor (phase 4). Phases 1–3 last for about 30 s and phase 4, 15–20 min. The severity of seizures increases with age, reaching a peak at around 2–3 months of age.

### Experimental groups and sound stimulations

Thirty-eight adult hamsters (19 GASH/Sal + 19 wild-type Syrian hamsters, 80–90 days old) were used. Experimental groups were as follows: resting control (not stimulated) hamsters (RC, *n* = 4 for qRT-PCR, n = 4 for Western blot, and n = 3 for immunohistochemistry), sound-stimulated control hamsters (SC, *n* = 4 for qRT-PCR and n = 4 for western blot), resting epileptic GASH/Sal hamsters (not stimulated) (RE, *n* = 4 for qRT-PCR, *n* = 4 for western blot, and *n* = 3 for immunohistochemistry), and sound-stimulated epileptic GASH/Sal hamsters (SE, *n* = 4 for qRT-PCR and *n* = 4 for western blot). Both SC and SE groups were exposed to white noise (1–37 KHz, 30–80 dB, 10 s) twice a day (with a 4–5-h interval) for 5 days. This protocol was chosen based on previous evidence from our laboratory to ensure the detection of significant variations in protein levels by Western blotting (23, 24).

### Immunohistochemistry

RC and RE hamsters were analyzed by microscopy (3 animals per group). After sacrifice, whole brains were removed from the skull, fixed by immersion for 10 min in buffered formalin and embedded in paraffin, using a Tissue Embedding System apparatus (TES99; Medite, Hanover, Germany). A 14 μm brain tissue sections were obtained with a microtome, deparaffinized at 60°C and rehydrated. Heat-induced epitope retrieval in 10 mmol L-1 citrate buffer was used at pH 6.0 for NKCC1 detection, and at pH 9.0 for GAD-67 detection. Sections were then immunohistochemically labeled with the standard avidin–streptavidin method using an automated platform (Dako autostainer; Dako, Glostrup, Denmark). Primary antibodies used were a monoclonal IgG antibody raised against a recombinant GAD-67 protein (Anti-GAD 67Antibody, MAB5406, clone 1G10.2, Merck KGaA, Darmstadt, Germany) and a monoclonal IgG antibody against the carboxy-terminus fragment (MET-902 to SER-1212) of the human NKCC1 protein as antigen (anti-NKCC1 contransporter, T4-c, Developmental Studies Hybridoma Bank, University of Iowa, Iowa City, USA), at a 1:1000 and 1:750 dilution, respectively. Diaminobenzidine was used as chromogen. Additional sections were processed as negative controls, omitting the incubation with the primary antibody.

Stained sections were observed using a Zeiss Axio Imager.M2 microscope (Carl Zeiss, Oberkochen, Germany). For image acquisition, Zen 2.6 software was utilized, while Image J was employed for image analysis. To measure the extent and intensity of staining, the parameter “integrated optical density” was used. For this densitometric analysis, measurements were limited to pixels exceeding a predetermined threshold level of staining intensity. A minimum of three hamsters from each experimental condition were included, with two coronal sections per animal and three to six regions of interest (ROIs) per section quantified. Only sections with matching coordinates were compared.

### Tissue sampling for molecular biology studies

Subjects from both sound-stimulated groups (SC and SE) were sacrificed immediately after the last stimulation (SC group) or last seizure (SE group). RC and RE hamsters were sacrificed on the same day without any extra manipulation. Animals were anesthetized (ketamine, 75 mg/kg + xylazine, 10 mg/kg), their brains quickly removed and dissected on ice, and the following brain areas were collected: cerebral cortex, hippocampus, hypothalamus, inferior colliculus, and pons-medulla oblongata. Brain tissue was immediately weighed after dissection and frozen on dry ice. Samples were kept at −80°C until assayed.

### Western blotting and antibodies

Four animals from each group were used for Western blot analyses. Brain regions were homogenized using a Polytron homogenizer (Kinematica AG, Lucerne, Switzerland) in extraction buffer (50 mM Tris HCl pH 7.4, 1% IGEPAL, 150 mM NaCl, 1 mM EDTA, 1 mM PMSF, 1 μg/μl leupeptin, and 1 mM NaF). Samples were fractionated by SDS-PAGE using the Mini-PROTEAN III Gel Electrophoresis System and transferred onto Hybond ECL nitrocellulose membranes (Amersham) for immunodetection, as previously described (25). NKCC1 was detected using mouse anti-NKCC1 (T4) monoclonal primary antibody (the same one used for immunohistochemistry), diluted to 1:300. GAD67 was detected using mouse anti-GAD67 monoclonal primary antibody (the same one used for immunohistochemistry), diluted to 1:1000. Monoclonal anti-alpha tubulin antibody (T5168, SIGMA) was diluted to 1:5000 and employed as loading control. Horseradish peroxidase-conjugated antibody against mouse IgG (#32430, ThermoScientific) was diluted to 1:1000. Immunoblots were read on a luminescent image analyzer LAS-3000 UV mini (Fujifilm, Tokyo, Japan), and densitometric analysis was performed using Quantity One Analysis Software (BioRad Laboratories, Hercules, CA. NKCC1 and GAD67 band optical densities were measured by densitometry, normalized to housekeeping alpha-tubulin protein band density and expressed as arbitrary density units.

### Quantitative reverse transcription PCR

Samples for qRT-PCR analysis were obtained from four hamsters from each experimental group. RNA was isolated using the RNeasy Minikit (Qiagen #74104) and treated with RNase-free DNase I, according to the manufacturer's instructions. Purified RNA was used for cDNA synthesis using RevertAid First-Strand cDNA Synthesis Kits (Thermo Scientific #K1622). The expression of *Slc12a2* and gad1 mRNA relative to gapdh mRNA (employed as housekeeping control) was determined using the 2–ΔΔCt method (26) with the following pairs: *Slc12a2* Fw forward primer 5′- CCACGATGAGCTGGAAAAGG-3′, *Slc12a2* Rv reverse primer 5′- CGCCTTTGATCCAGCCAAAC-3′, gad1Fw forward primer 5′-GCGCCTTTAGGGAGAAGCA-3′, gad1Rv reverse primer 5′-CCGGGTCACCGTTTTCAC-3′, gpadhFw forward primer 5′-CCATCTTCCAGGAGCGAGATC-3′, and gapdhRv reverse primer 5′-CCATCTTCCAGGAGCGAGATC-3′. PCR analysis was carried out with 1 μl of cDNA as a template in a reaction volume of 10 μl containing 5 μl of Power SYBR Green PCRMaster Mix (Thermo Fisher Scientific) and 200 nM of each primer. Thermocycling included an initial denaturation step at 95°C for 10 min, followed by 40 cycles consisting of 15 s denaturation at 95°C for 60 s, and a combined annealing and extension step at 60°C for 40 s. The PCR products and their dissociation curves were detected with an ABI PRISM 7500Fast real-time PCR system (Life Technologies).

### Statistical analysis

Data are expressed as means ± S.E.M. Two-way analysis of variance was used when required. ANOVA factors to assess group means were: strain (levels: wild-type control and GASH/Sal) × sound stimulation (levels: stimulation and no stimulation). ANOVA analysis was followed by the multiple range Holm-Sidak for post hoc assessment of individual means.

## Results

### Sound stimulation

There is evidence suggesting that KCC2 expression is altered in epileptic GASH/Sal hamsters (23). To determine whether NKCC1 also contributes to the Cl^−^ imbalance associated with epilepsy in these rodents, both GASH/Sal and wild-type hamsters were subjected to a sound stimulation procedure that was optimized in previous studies (23, 24). Although no spontaneous seizures were observed in either animal group, the sound stimulation resulted in full 4-phase generalized seizures in all epileptic GASH/Sal hamsters tested, while no behavioral changes were detected in wild-type control hamsters. No animals died due to sound-stimulated seizures.

### NKCC1 protein expression

**Figure 1:**
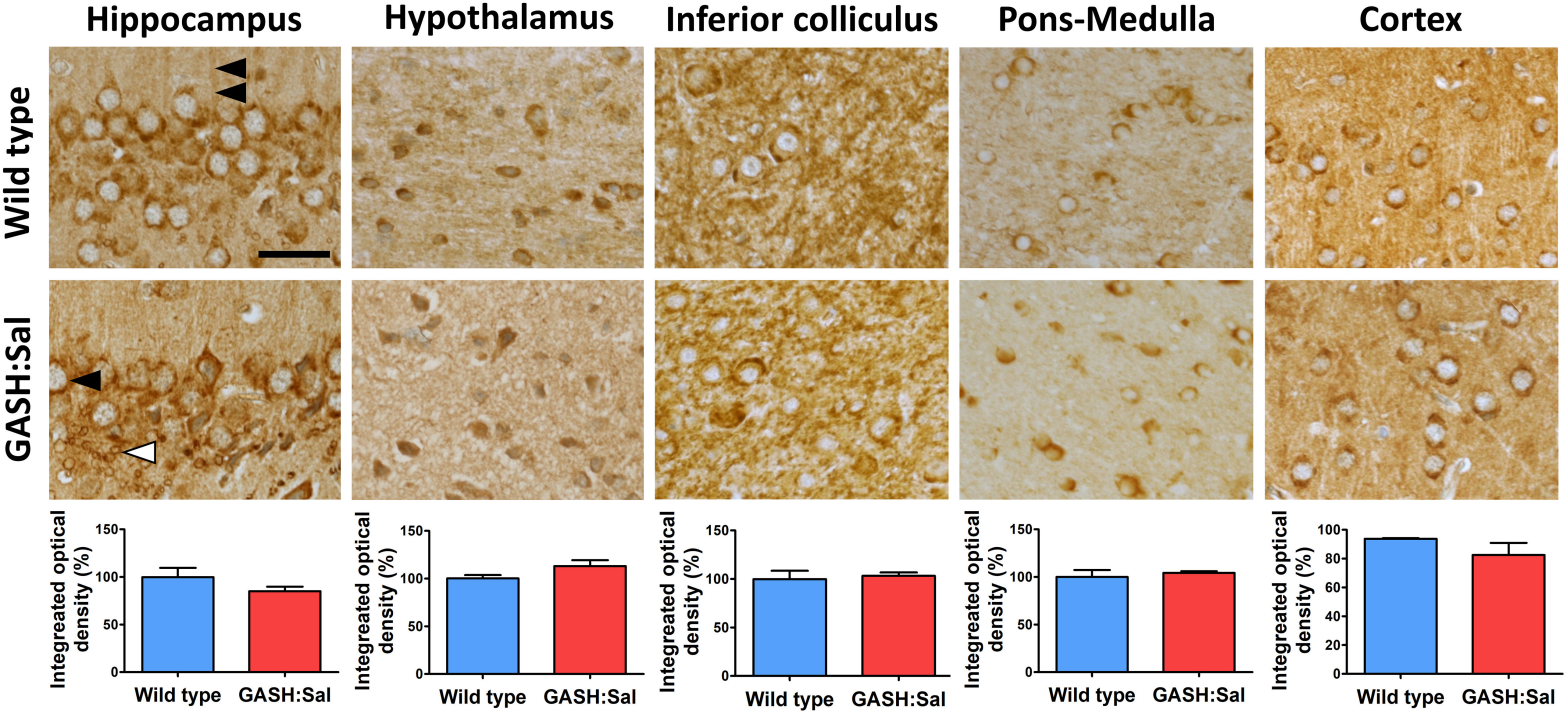
Brain NKCC1 immunoreactivity in GASH/Sal epileptic hamsters. NKCC1 immunostaining was evaluated in several brain regions of 3 epileptic GASH/Sal hamsters and compared to 3 wild-type controls. Microscopy images were obtained from paraffin-embedded brain sections containing hippocampus, hypothalamus, inferior colliculus, pons-medulla oblongata and cortex. The immunohistochemistry technique employed was the avidin–biotin-peroxidase complex method with 3′ 3-diaminobenzidine chromogen. Labeling was observed in dendrites (double arrowhead), cell somas resembling neurons (black arrowhead) and in glial cells (white arrowhead), based on their size and distribution. Scale bar, 25 μm. The graphs illustrate the integrated optical density as a measure of the extent and intensity of NKCC1 immunostaining in each of the analyzed brain regions. Values are expressed as percentage of the immunoreactivity in wild-type hamsters. A *t*-test was used for statistical analysis. Images show that the pattern of NKCC1 immunoreactivity in GASH/Sal hamsters is similar to that of wild-type controls.

To investigate the potential dysregulation of brain NKCC1 in epileptic hamsters, we first compared brain NKCC1 immunoreactivity in unstimulated GASH/Sal hamsters with wild-type controls through microscopy. Since seizures in GASH/Sal hamsters are produced by sound, the brain areas selected for this study included the hippocampus, hypothalamus, inferior colliculus, pons-medulla and cortex, as they are involved in brain auditory processing and/or implicated in epileptogenesis (22–24, 27, 28). The micrographs obtained revealed widespread NKCC1 staining throughout the brain (Figure 1), consistent with previous findings (29–31). Although NKCC1-positive labeling was present in all brain areas examined, the pattern of NKCC1 expression exhibited highly specific regional differences. NKCC1-positive cells displayed similarities to both neurons and glia in terms of size and morphology. However, a definitive identification of the cell-types involved would require co-localization studies using specific neuronal, astrocyte, microglia, and oligodendrocyte markers. Some positive granules in neural cytoplasm may correspond to lipofuscin inclusions, previously identified by other authors (32). Comparison of NKCC1 immunoreactivity between GASH/Sal and wild-type brain sections revealed no obvious signs of altered NKCC1 expression in the epileptic strain in terms of staining intensity or distribution pattern, which was confirmed by the densitometric analysis of NKCC1 immunoreactivity in all brain regions examined.

**Figure 2:**
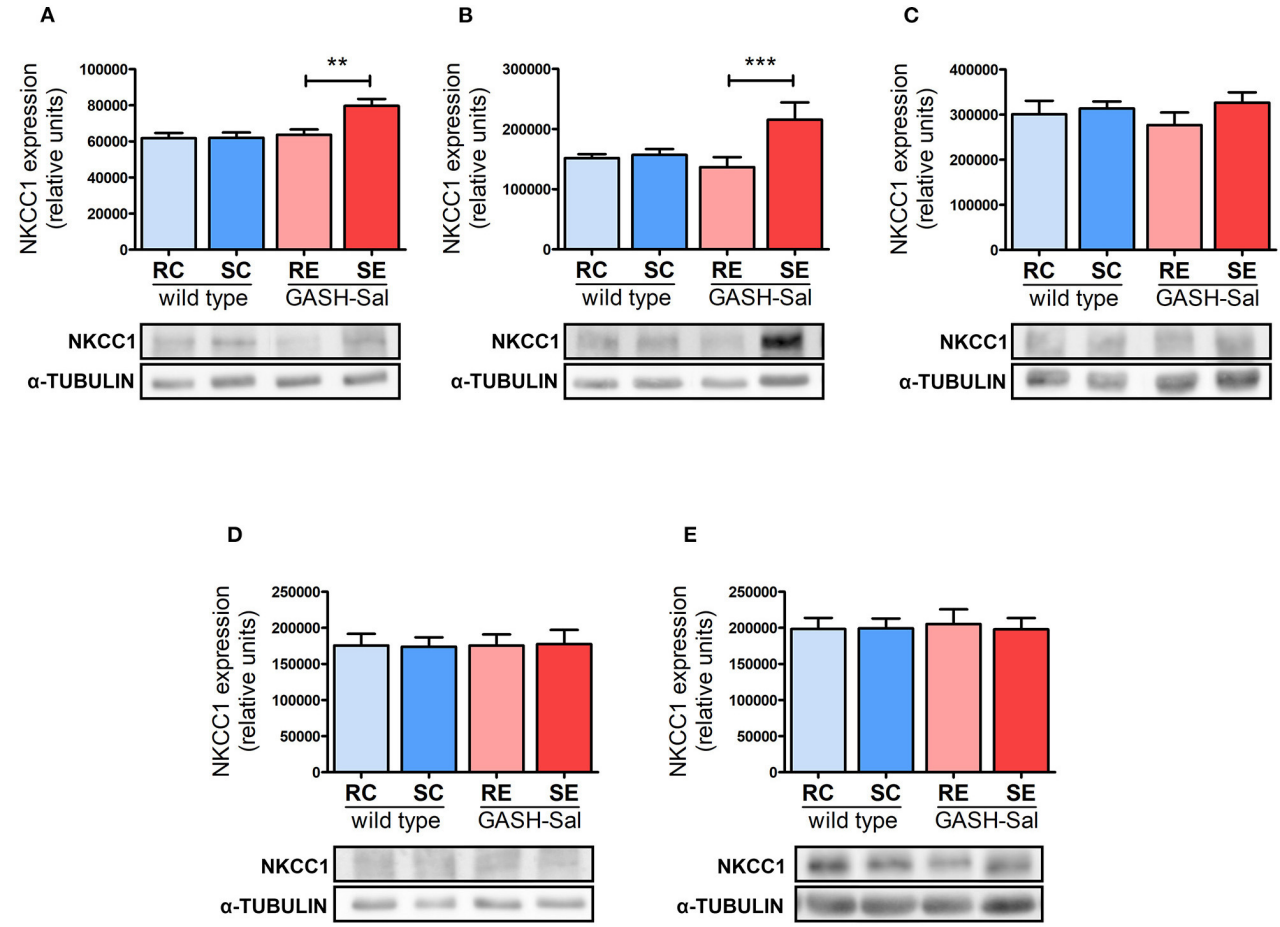
Effects of seizure induction on NKCC1 protein expression. Brain levels of NKCC1 protein were evaluated by Western blotting in 4 epileptic GASH/Sal and 4 wild-type hamsters. Brain regions analyzed include hippocampus **(A)**, hypothalamus **(B)**, inferior colliculus **(C)**, pons and medulla oblongata **(D)**, and cortex **(E)**. NKCC1 protein expression was determined both at rest and after repeated sound-inducing seizures. α-Tubulin was included as loading control. Representative blots for each experimental condition are shown. Data correspond to means ± S.E.M. from 4 animals. Band densities were normalized to alpha-tubulin protein and expressed as arbitrary density units. Results were analyzed using a two-way ANOVA (factors: “strain” and “sound stimulation”) followed by the multiple range Holm-Sidak test. **p < 0.01, ***p < 0.001. RC, resting control hamsters; SC, sound-stimulated control hamsters; RE, resting epileptic hamsters; SE, sound-stimulated epileptic hamsters. Seizures upregulate NKCC1 protein in specific brain areas.

After the qualitative assessment of immunoreactivity through imaging, we quantified the amount of NKCC1 protein in GASH/Sal vs. wild-type hamsters using Western blotting. Densitometric analysis of NKCC1 from the hippocampus, hypothalamus, inferior colliculus, pons-medulla, and cortex showed comparable basal expression levels in both hamster strains (RC vs. RE in Figure 2), confirming our earlier microscopy analysis.

Next, we investigated the potential effects of seizure induction and found that repeated sound stimulation had notable effects. Changes were exclusively detected in the GASH/Sal strain (RE vs. SE in Figure 2), as wild-type animals remained unaffected (RC vs. SC in Figure 2). Furthermore, NKCC1 regulation by seizures exhibited a clear regional specificity. Hence, convulsions led to a statistically significant increase in NKCC1 protein expression in the hippocampus and hypothalamus, with values rising by 30 and 50%, respectively. Meanwhile, no alterations were detected in the inferior colliculus, pons-medulla oblongata or cortex.

### NKCC1 gene expression

**Figure 3:**
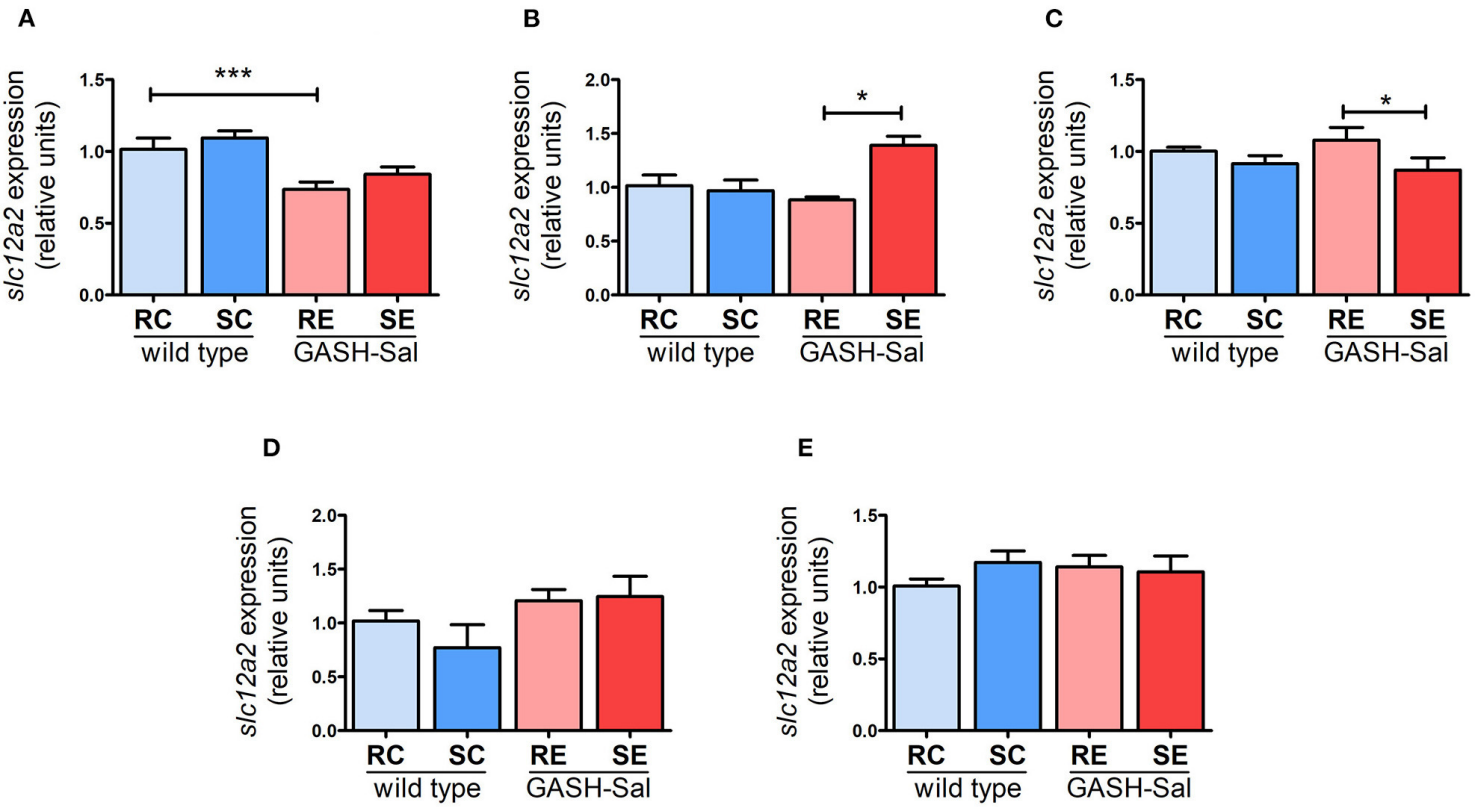
Changes in NKCC1 transcription induced by seizures. NKCC1 mRNA expression in 4 wild-type and 4 epileptic GASH/Sal hamsters was analyzed by qRT-PCR. Brain regions analyzed include hippocampus **(A)**, hypothalamus **(B)**, inferior colliculus **(C)**, pons-medulla oblongata **(D)**, and cortex **(E)**. NKCC1 mRNA expression was determined both at rest and after repeated sound-inducing seizures. The values represent the average of four animals carried out in triplicate. The expression of *Slc12a2* mRNA was normalized using gapdh as a housekeeping gene. The data are presented as relative expression compared to the control group (resting control hamsters). Results were analyzed using a two-way ANOVA (factors: “strain” and “sound stimulation”) followed by the multiple range Holm-Sidak test. *p < 0.05, ***p < 0.001. RC, resting control hamsters; SC, sound-stimulated control hamsters; RE, resting epileptic hamsters; SE, sound-stimulated epileptic hamsters. Changes in transcription affect NKCC1 expression in specific brain areas.

We next investigated whether the upregulation of NKCC1 protein observed in specific brain regions of the GASH/Sal hamster after repeated audiogenic seizures was post-transcriptional or, alternatively, it involved changes in gene expression. Quantification of *NKCC1* gene expression using qRT-PCR revealed that *Slc12a2* mRNA levels were comparable between unstimulated GASH/Sal and wild-type hamsters in all brain areas examined, except for the hippocampus, which exhibited a significant reduction in *Slc12a2* expression in the GASH/Sal hamster under resting conditions (Figure 3).

Moreover, the induction of seizures had a mixed impact on the regulation of the *Slc12a2* gene in GASH/Sal hamsters (Figure 3). NKCC1 mRNA was significantly upregulated particularly in hypothalamus (by 40%), implying that the detected increase in protein expression in this region was due to transcriptional regulation. Conversely, there were no changes in *Slc12a2* expression in the hippocampus, even though NKCC1 protein levels rose after seizure induction in this brain region. Additionally, a modest reduction in *Slc12a2* levels (by 16%) was observed in the inferior colliculus, without any noticeable changes in NKCC1 protein levels. These findings suggest that the effect of repeated seizures on NKCC1 expression can vary between transcriptional and post-transcriptional regulation, depending on the specific brain region analyzed.

### GAD67 protein expression in GASH/Sal vs. wild-type hamsters

**Figure 4:**
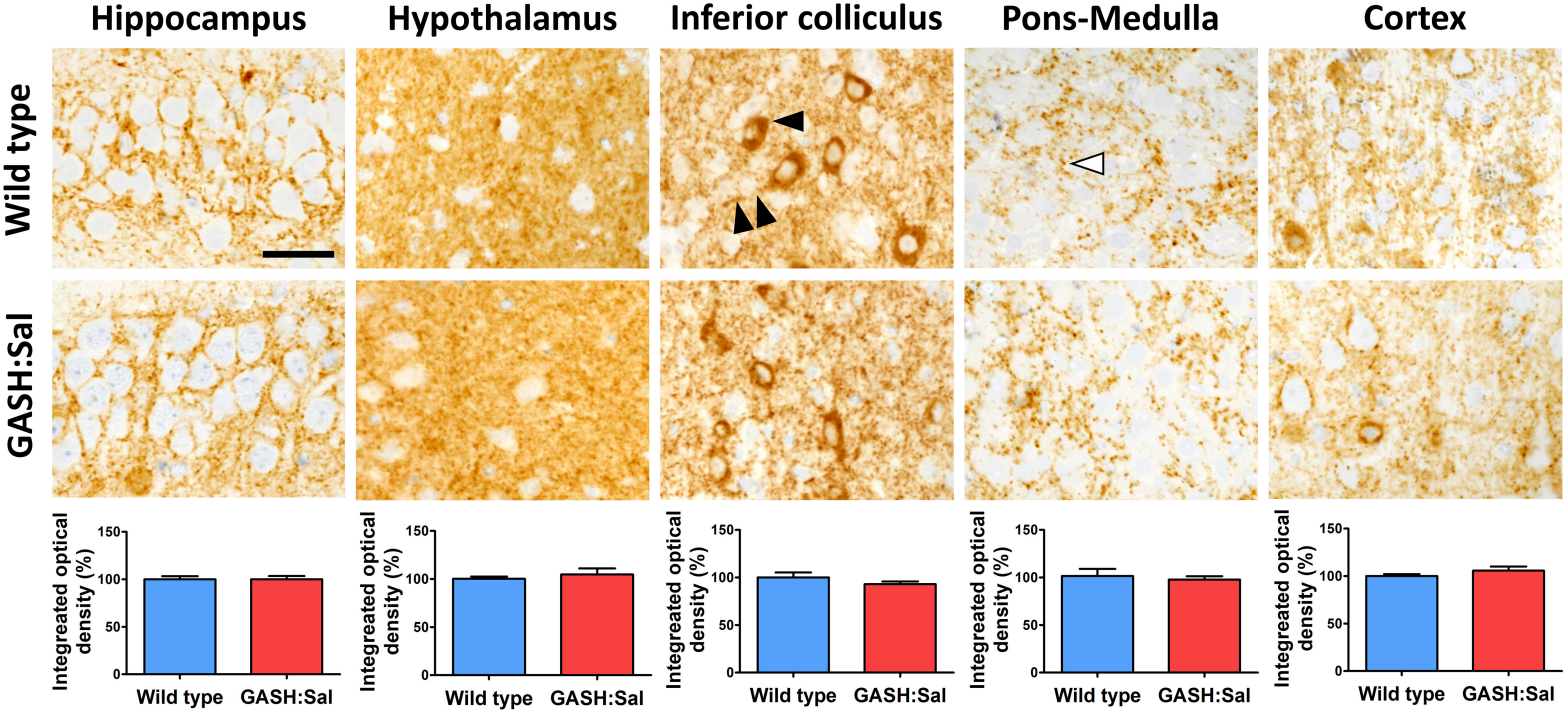
Brain GAD67 immunoreactivity in GASH/Sal epileptic hamsters. GAD67 immunostaining was evaluated in several brain regions of 3 epileptic GASH/Sal hamsters and compared to 3 wild-type controls. Microscopy images were obtained from paraffin-embedded brain sections containing hippocampus, hypothalamus, inferior colliculus, pons-medulla oblongata and cortex. The immunohistochemistry technique employed was the avidin–biotin-peroxidase complex method with 3′ 3-diaminobenzidine chromogen. Immunostaining was found in neurites (double arrowhead), somas of neurons (black arrowhead), and small puncta (white arrowhead). Scale bar, 25 μm. The graphs illustrate the integrated optical density as a measure of the extent and intensity of GAD67 immunostaining in each of the analyzed brain regions. Values are expressed as percentage of the immunoreactivity in wild-type hamsters. A *t*-test was used for statistical analysis. The pattern of GAD67 immunoreactivity in GASH/Sal hamsters is similar to that of wild-type controls.

Previous reports have indicated dysregulation of GABA neurotransmission in brains areas affected by epilepsy (12). As changes in the expression of GAD67 (involved in GABA synthesis) have been linked to alterations in GABA release (33), we investigated whether changes in NKCC1 expression induced by convulsion could be associated with changes in GAD67 levels in the GASH/Sal model of epilepsy. We first compared basal GAD67 protein labeling in GASH/Sal hamsters with their wild-type counterparts using microscopy. The same brain regions studied for NKCC1 expression were examined: hippocampus, hypothalamus, inferior colliculus, pons-medulla and cortex. As shown in Figure 4, the microscopy analysis of hamster brains revealed the broad distribution of GAD67 immunoreactivity in unstimulated conditions. Specific labeling was present in all brain regions examined, with intense GAD67 immunoreactivity observed in neuronal somas and neurites. Additionally, significant staining was found in small puncta displaying a size and distribution consistent with synaptic structures. Although GAD67 staining showed a characteristic pattern in each brain area examined, no apparent differences in GAD67 immunoreactivity between GASH/Sal and wild-type animals were observed, which was confirmed by the densitometric analysis of GAD67 immunoreactivity.

Western blotting was used to further investigate whether the GASH/Sal strain presented a distinctive brain expression pattern for GAD67 protein with respect to their wild-type counterparts. However, densitometry analysis of GAD67 bands confirmed that GAD67 protein expression was similar in both genotypes in all brain regions examined, which confirms the lack of differences observed in microscopy images. This Western blot analysis also revealed absence of significant changes in GAD67 protein expression in brain areas of both hamster strains after seizure induction (Supplementary Figure 1).

### GAD67 mRNA induction

**Figure 5:**
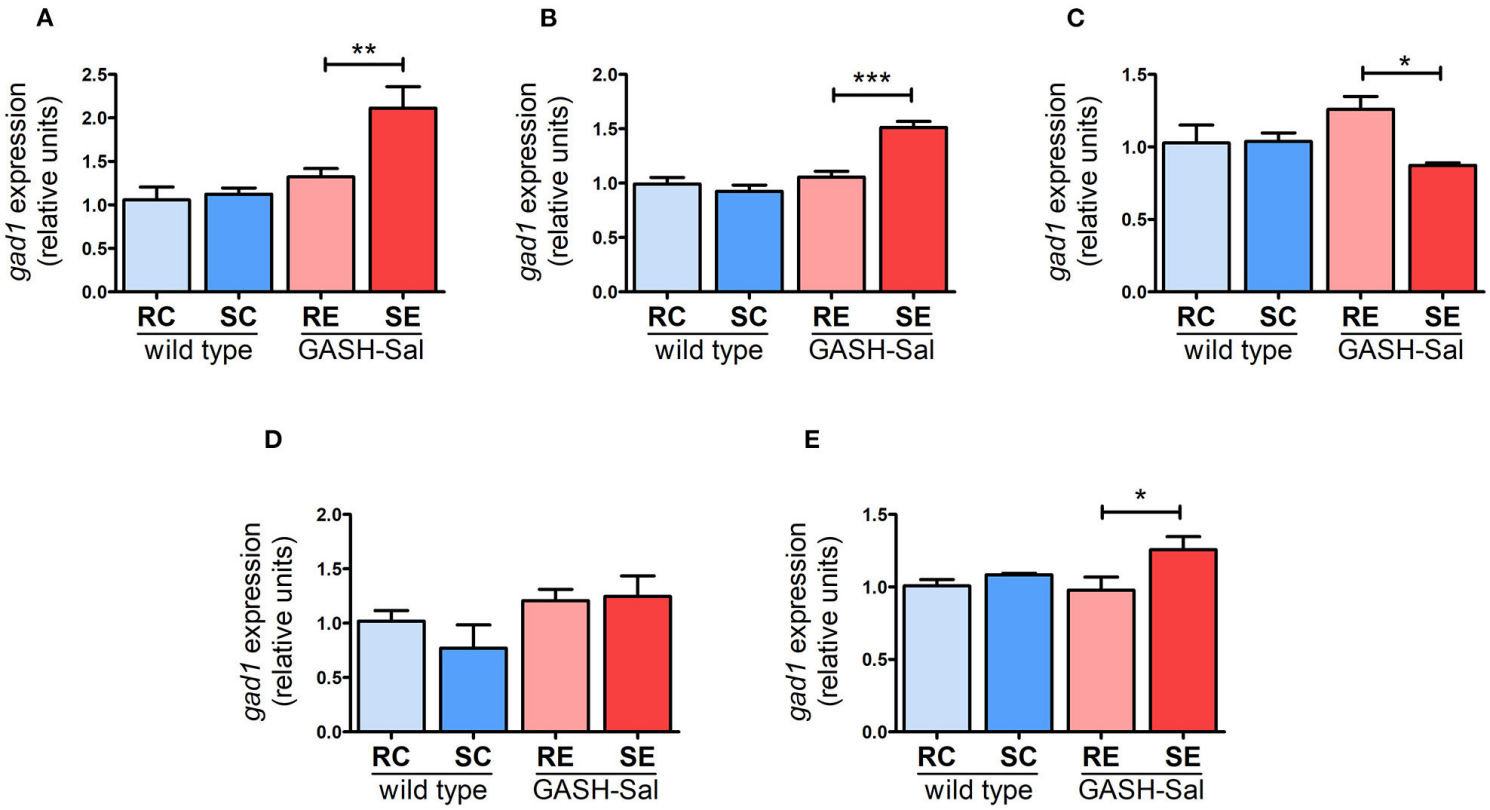
Effects of seizures on *GAD67* gene expression. GAD67 mRNA levels were analyzed using qRT-PCR in 4 epileptic GASH/Sal and 4wild-type hamsters. Brain regions analyzed include hippocampus **(A)**, hypothalamus **(B)**, inferior colliculus **(C)**, pons and medulla oblongata **(D)**, and cortex **(E)**. GAD67 mRNA expression was determined both at rest and after repeated sound-inducing seizures. The values represent the average of four animals carried out in triplicate. The expression of GAD67 mRNA was normalized using gapdh as a housekeeping gene. The data are presented as relative expression compared to the control group (resting control hamsters). Results were analyzed using a two-way ANOVA (factors: “strain” and “sound stimulation”) followed by the multiple range Holm-Sidak test. *p < 0.05, **p < 0.01, ***p < 0.001. RC, resting control hamsters; SC, sound-stimulated control hamsters; RE, resting epileptic hamsters; SE, sound-stimulated epileptic hamsters. GAD67 transcription is regulated by seizures in specific brain areas.

As we found no evidence that the seizure induction protocol applied modulates GAD67 protein expression in the GASH/Sal strain, our last experiments focused on determining whether changes in NKCC1 observed in epileptic hamsters could be linked to the regulation of GAD67 at the transcriptional level. The collected qRT-PCR data showed that *Gad1* mRNA levels were similar in GASH/Sal and wild-type hamsters across all brain regions examined. However, seizure induction significantly upregulated *Gad1*mRNA levels in the hippocampus (by 55%), hypothalamus (by 44%), and cortex (by 27%). Additionally, we observed a decrease in inferior colliculus (by 35%), while no changes were found in the pons-medulla oblongata (Figure 5). Therefore, these findings suggest that GAD67 is broadly regulated by seizures at the transcriptional level in GASH/Sal hamsters. According to previous observations, the upregulation of *Gad1*mRNA levels indicates changes in GABA release consistent with the potentiation of GABA neurotransmission (33). Furthermore, the substantial increase in GAD67 mRNA expression detected in the hippocampus and hypothalamus, along with the observed upregulation of NKCC1 expression, could potentially contribute to the exacerbation of epilepsy in these brain regions of the GASH/Sal hamster model. Thus, altogether, these findings support the notion that seizure-triggered changes in NKCC1 expression may contribute to altered GABA neurotransmission in epileptic hamsters.

## Discussion

The present study focuses on analyzing NKCC1 expression in the GASH/Sal epileptic hamster strain using complementary techniques such as microscopy, Western blotting, and RT-PCR. The brain areas analyzed were selected based on previous studies that established their implication in audiogenic seizures in rodents (22–24, 27, 28). Additionally, the hippocampus has been identified as a common focus of seizures, likely due to its high electrical excitability. The broad NKCC1 expression pattern observed complements the similarly widespread expression of KCC2 reported in the brain (29–31, 34). KCC2 expression in GASH/Sal hamsters was examined by our team in a previous work (23). On the contrary, to our knowledge, the expression profile of NKCC has not been examined in hamsters prior to this study, despite the fact that NKCC1 has been reported in other rodent species such as mice and rats, in brain regions that include the cortex, hippocampus, cerebellum, and pons-medulla.

Our experiments revealed that sound-triggered seizures significantly increased NKCC1 expression in specific brain regions of epileptic GASH/Sal hamsters, whereas no significant differences were observed in NKCC1 protein and mRNA levels between GASH/Sal and wild-type hamsters under unstimulated conditions. An increase in NKCC1 expression has been reported in the hippocampus in a post-traumatic seizures mouse model (35). This is consistent with our current findings, even though seizures in this just mentioned model also upregulated NKCC in the brain cortex. Our results are also in line with a previous study demonstrating that convulsions induced by the same sound stimulation protocol also modulate the expression of KCC2 in GASH/Sal hamsters (23). In this just mentioned study, KCC2 protein levels were significantly lower in several brain regions of GASH/Sal hamsters compared to wild-type animals even at rest. In contrast, our results showed that NKCC1 regulation was only convulsion-dependent. Taken together, these findings suggest that seizures can simultaneously upregulate NKCC1 and downregulate KCC2 expression in specific brain areas such as the hypothalamus. This dual regulation of cation-Cl^−^ cotransporters by seizures may cause an accumulation of intracellular Cl^−^ ions and facilitate GABA excitatory neurotransmission (29, 30). However, the changes in NKCC1 and KCC2 expression observed in GASH/Sal epileptic hamsters differ by brain region; thus, it is unlikely that they contribute to the development of a GABA excitatory tone in the entire brain.

Indeed, the upregulation of NKCC1 expression found in specific brain areas of epileptic hamsters could have significant implications for GABA neurotransmission. This is due to the fact that type A GABA ionotropic receptors (GABA_A_R) are ligand-gated Cl^−^ ion channels (36) whose inhibitory or excitatory effects depend on Cl^−^ concentrations on both sides of the plasma membrane in the postsynaptic neuron (8). In adults, GABA release generally causes Cl^−^ influx through the GABAA receptor, leading to neuronal hyperpolarization and decreased excitability. However, during development, GABA action is mainly excitatory, which is facilitated by the high expression of brain NKCC1 (37). In our experiments, the upregulation of NKCC1 levels observed in certain brain areas of the epileptic hamsters would lead to an increase in intracellular Cl^−^ levels. GABA release in this scenario could cause Cl^−^ outward currents and depolarization of the postsynaptic neuron (8). Therefore, the upregulation of NKCC1 found in the epileptic hamster in our experiments resembles the situation previously described for immature animals, in which the high expression of brain NKCC1 is considered a key factor for GABA excitatory neurotransmission (10). Thus, our findings support the previously proposed hypothesis that seizures may reverse the adult NKCC1/KCC2 balance, favoring the excitatory GABA neurotransmission observed in development. Moreover, the increased excitatory tone observed in adults due to the upregulation of NKCC1 could increase their susceptibility to epilepsy and exacerbate seizures.

Interestingly, the gene encoding NKCC1 produces two splice variants, NKCC1a and NKCC1b, whose expression is predominant in glial cells and neurons, respectively. There is evidence indicating that the neuronal isoform NKCC1b is downregulated during development so that glial NKCC1a is the predominant isoform in the adult brain (9). Our experiments examining NKCC1 immunoreactivity detected NKCC1-positive cells in both control and epileptic hamsters, and images are suggestive of NKCC1 expression in both glial cells and neurons. However, identification of the specific cell types showing NKCC1 labeling would involve co-localization studies using specific neuronal, astrocyte, microglia and oligodendrocyte markers, which is beyond the scope of our study. In addition, it is important to note that the observed increase in NKCC1 expression detected through Western blotting in our model could potentially correspond to both glial cells and/or neurons. Given the pivotal role of glial cells, particularly astrocytes, in maintaining ion homeostasis in the brain, it is evident that both types of effects may have significant implications for neurotransmission and epilepsy (38). In support of this notion, NKCC1 inhibitors such as bumetadine, which target both neurons and glial cells expressing NKCC1, exhibit significant anti-epileptic effects (39).

We measured GAD67 mRNA levels to identify the brain areas affected by seizures in terms of GABA neurotransmission. GAD67 is known to regulate GABA synthesis to maintain basal neurotransmitter levels in GABA neurons and play a crucial role in the maturation of the GABAergic system during development. Mice lacking GAD67 have significantly reduced cerebral GABA levels and die perinatally, highlighting the essential role of GAD67 in maintaining GABA neurotransmission (3). The association between GAD67 expression and the regulation of GABA neurotransmission is maintained in the adult brain. In this regard, disruption of GAD67 expression reduced GABA levels in hippocampal neurons (4). Additionally, there is evidence to suggest that changes in GABA neurotransmission may affect GAD67 expression (33). As in our experiments, this just mentioned report involved measuring changes in GAD67 mRNA levels as an indirect but more suitable approach to identify changes in GABA neurotransmission than direct evaluation of GABA release.

Our findings indicate that GAD67 transcription is upregulated in several brain areas of epileptic hamsters in response to convulsions. This is in line with previous reports that have shown a correlation between changes in GAD67 activity and GABA release in epileptic brains. In particular, GAD67 expression has been found to increase in the hippocampus of rats after sustained epileptic seizures (40), and in the hippocampus of patients with mesial temporal lobe epilepsy (14). It has been proposed that this upregulation of GAD67 expression converts excessive glutamate into GABA as a compensatory mechanism to boost GABA inhibitory neurotransmission. Additionally, GAD67 expression was found to increase in a knockout mouse model of epilepsy, which was associated with enhanced inhibitory neurotransmission in brain regions with epileptiform activity (15). Another study found that adenoviral transduction of GAD67 in hippocampus of epileptic mice significantly attenuated convulsions, further supporting the idea that increased GAD expression can counterbalance hyperexcitation (41). Interestingly, our experiments showed that GAD67 regulation by convulsions had specificity, affecting brain regions where we also observed upregulated NKCC1 expression caused by seizures, such as the hippocampus and hypothalamus. This coordination between GAD67 and NKCC1 regulation in epileptic hamsters may not be coincidental, as GAD67 upregulation could be compensating for the hyperexcitation caused by the increase in NKCC1 expression. Finally, it should be mentioned that we observed changes in GAD67 mRNA without corresponding changes in GAD67 protein. This is not surprising, since changes in protein levels can be delayed due to factors such as translation kinetics, protein folding and post-translational modifications; thus, protein regulation may not perfectly correlate with mRNA changes at a specific time point. Additionally, possible technical limitations in protein detection or a rapid GAD67 protein degradation rate within our experimental system, may contribute to overlooking changes in the expression of this protein.

The findings of altered NKCC1 and GAD67 expression in response to seizures in the GASH/Sal strain support the use of this model to investigate the various factors that impact the GABAergic system in epilepsy. The GASH/Sal strain is a natural model of genetic audiogenic epilepsy (19), validated by ictal electroencephalogram recordings (Carballosa-Gonzalez) and the similarity of GASH/Sal seizures to other established models (20). The involvement of GABA neurotransmission in GASH/Sal epilepsy is supported by the dysregulation of GABA_A_R found in this model (23). Additionally, evidence indicates that the KCC2 cotransporter is altered in several brain regions of the GASH/Sal hamster, both at rest and after repeated seizure induction (23).

Our results suggest that seizures may worsen convulsive episodes by increasing NKCC1 expression, highlighting the potential therapeutic value of manipulating NKCC1 in epilepsy. While benzodiazepines, barbiturates and other antiepileptic drugs enhance GABA-mediated inhibition by targeting GABA_A_Rs (13), our findings, along with previous research (23), indicate that cation-chloride cotransporters are also modulated in epilepsy in a manner that likely limits GABA inhibitory neurotransmission. As a result, pharmacological agents that reduce NKCC1 activity and/or increase KCC2 expression should be investigated as potential antiseizure drug alternatives to those targeting GABA_A_R.

In conclusion, this study reveals that the NKCC1 cotransporter is sensitive to seizures in specific brain regions and supports the upregulation of NKCC1 as a contributing factor to the dysregulation of GABA neurotransmission in epileptic hamsters. Our findings emphasize the need to further explore the therapeutic potential of targeting NKCC1 in epilepsy.

## Data availability statement

The raw data supporting the conclusions of this article will be made available by the authors, without undue reservation.

## Ethics statement

The animal study was reviewed and approved by the Animal Experimentation Committee of the University of Castilla - La Mancha (Ref. No. 1205_03). All experimental procedures were in accordance with the Declaration of Helsinki and the Guidelines of the Directive 2010/63/EU of the European Parliament and of the Council.

## Author contributions

Conceptualization: CC, PT, DL, LM, and JE. Formal analysis: CC, J-MB-F, PT, and JA-A. Investigation: J-MB-F, JA-A, and CC. Verification of the underlying data: J-MB-F, JA-A, CC, and PT. Writing–original draft preparation: CC, J-MB-F, PT, DL, LM, and JE. Writing–review and editing: CC, PT, DL, LM, and JE. Supervision: CC, JE, JA-A, and PT. All authors had full access to all of the data in the study, accepted responsibility for submission of the work for publication, contributed to the article, and approved the submitted version.

## Supplementary material

Supplementary Figure 1 Brain expression of GAD67 protein in GASH/Sal epileptic hamsters. Brain levels of GAD67 protein were evaluated by Western blotting in four wild-type and four epileptic GASH/Sal hamsters. Brain regions analyzed include the hippocampus **(A)**, hypothalamus **(B)**, inferior colliculus **(C)**, pons and medulla oblongata **(D)**, and cortex **(E)**. GAD67 protein expression was determined both at rest and after repeated sound-inducing seizures. a-Tubulin was included as loading control. Representative blots for each experimental condition are shown. Data correspond to means ± S.E.M. from 4 animals per experimental group. Band densities were normalized to alpha-tubulin protein band density and expressed as arbitrary density units. Results were analyzed using a two-way ANOVA (factors: "strain" and "sound stimulation") followed by the multiple range Holm-Sidak test. ^***^p < 0.001. RC, resting control hamsters; SC, sound-stimulated control hamsters; RE, resting epileptic hamsters; SE, sound-stimulated epileptic hamsters. No changes in GAD67 protein levels could be detected as a result of seizures.
