# Supplementary figures and images for: Seizures regulate the cation-Cl− cotransporter NKCC1 in a hamster model of epilepsy: implications for GABA neurotransmission
Source: Front Neurol. 2023 Jun 22;14:1207616. doi: 10.3389/fneur.2023.1207616 (PMC10338185; doi:10.3389/fneur.2023.1207616)

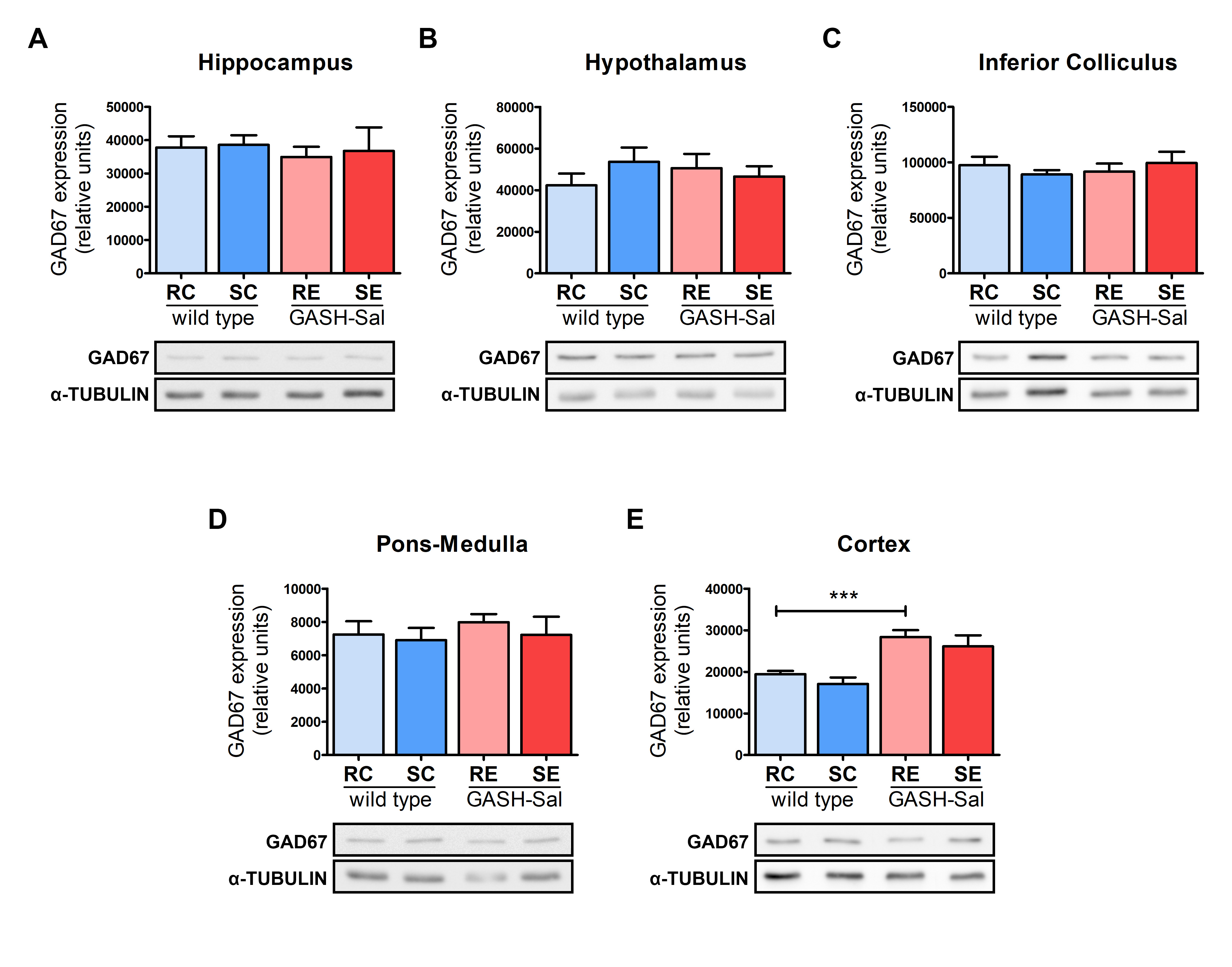

Supplement: Supplementary Figure 1 — Brain expression of GAD67 protein in GASH/Sal epileptic hamsters. Brain levels of GAD67 protein were evaluated by Western blotting in four wild-type and four epileptic GASH/Sal hamsters. Brain regions analyzed include the hippocampus (A), hypothalamus (B), inferior colliculus (C), pons and medulla oblongata (D), and cortex (E). GAD67 protein expression was determined both at rest and after repeated sound-inducing seizures. a-Tubulin was included as loading control. Representative blots for each experimental condition are shown. Data correspond to means ± S.E.M. from 4 animals per experimental group. Band densities were normalized to alpha-tubulin protein band density and expressed as arbitrary density units. Results were analyzed using a two-way ANOVA (factors: "strain" and "sound stimulation") followed by the multiple range Holm-Sidak test. ***p < 0.001. RC, resting control hamsters; SC, sound-stimulated control hamsters; RE, resting epileptic hamsters; SE, sound-stimulated epileptic hamsters. No changes in GAD67 protein levels could be detected as a result of seizures. [file Image_1.JPEG]
